# Indulgence and Long Term Orientation Influence Prosocial Behavior at National Level

**DOI:** 10.3389/fpsyg.2018.01798

**Published:** 2018-09-24

**Authors:** Qingke Guo, Zhen Liu, Xile Li, Xiuqing Qiao

**Affiliations:** ^1^Department of Psychology, Shandong Normal University, Jinan, China; ^2^Department of Psychology, Southwest University, Chongqing, China

**Keywords:** prosocial behavior, Hofstede’s cultural dimensions, indulgence versus restraint, long-term versus short-term orientation, individualism versus collectivism

## Abstract

The relationships between several Hofstede’s cultural dimensions and prosocial behavior at national level have been investigated by some studies. Yet the roles of indulgence versus restraint (IVR) and long-term versus short-term orientation (LTO), two newly established cultural dimensions, have received insufficient interest. This study aimed to investigate whether the World Giving Index (WGI), a national level measure of prosocial behavior (including donating, volunteering, and helping a stranger) provided by Gallup, was affected by IVR and LTO. The results suggested a positive link between IVR and WGI, and a negative link between LTO and helping a stranger. Culture values can in a great extend account for why prosocial behavior varies across countries. Further analysis revealed interactions among IVR, LTO, and individualism versus collectivism (IND). Simple slope analyses found that: (1) a higher level of IND could enhance the positive influence of IVR on prosocial behavior; (2) a lower level of IND could weaken the negative impact of LTO on prosocial behavior; (3) a higher level of IVR could weaken the negative effect of LTO on prosocial behavior.

## Introduction

People are willing to sacrifice their own interests, including time, energy, money, and even physical health, to benefit others or the society as a whole. These behaviors are called prosocial behavior, which includes sharing, formal and informal helping, charitable donation, and volunteering (Zwick and Fletcher, 2014; Henrich, 2015). In the attempt to understand why people conduct such behaviors, the role of culture has been revealed by more and more researchers. Previous literature found that the frequency and manifestations of prosocial behavior may be influenced by culture (e.g., Henrich, 2015). For example, Daniel et al. (2015) found that helping behavior varied across countries (i.e., Germany, Scotland United Kingdom, Israel, and Turkey) and was significantly related to values (Schwartz, 2010). In the abovementioned study, helping behavior was positively associated with self-transcendence, and was negatively associated with self-enhancement and openness to change (Daniel et al., 2015). Additionally, differences in parochial altruism or in-group preference have been observed between collectivist societies (i.e., Chinese) and individualist societies (i.e., United States; Henrich, 2015). Recently, several studies have investigated the relationships between Hofstede’s cultural dimensions and prosocial behavior at national level (Winterich and Zhang, 2014; Luria et al., 2015; Smith, 2015; Stojcic et al., 2016). For example, drawing on the data of giving around the world gathered by the Gallup organization in 2013, Smith (2015) found that countries with high scores of uncertainty avoidance usually had lower levels of prosocial behavior. In the 2013 Gallup survey, the percentage of volunteering participation was largest in Denmark (69%), a country whose uncertainty avoidance score was 23, and smallest in Greece (10%), a country whose uncertainty avoidance score was 112. Luria et al. (2015) also found that several Hofstede’s cultural dimensions and their interactions had considerable influences on nation-level prosocial behavior. However, there was insufficient interest in the roles of IVR and LTO, two newly established cultural dimensions. Can they also account for the differences in prosocial behavior across nations? This study aimed to investigate the roles of IVR and LTO, as well as their interactions with other cultural dimensions, in the prediction of prosocial behavior at national level.

### Hofstede’s Model of Cultural Values

Hofstede’s (1980) model of cultural values was developed as the results of a world-wide values survey conducted in 116,000 IBM workers from 40 countries/regions. The model was defined by a system of attitudes, beliefs, and values shared by individuals in a society or group. Hofstede initially identified four culture dimensions, namely (1) Power distance, indicating the extent to which the less powerful members of a culture group accept and expect that power is distributed unequally; (2) IND, which means how strong the individuals in a society are tied together or integrated into groups; (3) Uncertainty avoidance, stating a society’s tolerance for uncertainty and ambiguity; (4) Masculinity versus femininity, indicating the extent to which masculine values (e.g., achievement and material reward) relative to feminine values (e.g., cooperation and quality of life) are preferred in a society.

Subsequently, the fifth dimension (i.e., LTO) and the sixth dimension (i.e., IVR) were added to Hofstede’s model (Bond and Hofstede, 1989; Minkov and Hofstede, 2012). LTO originated from Bond’s Chinese Value Survey comparing students from 23 countries (see Hofstede and Bond, 1988), and was initially labeled by Bond as *Confucian Work Dynamism*. LTO indicates the time-orientation of a society. Societies located at the long-term pole prefer virtues oriented toward future reward, particularly perseverance, thrift, order of relation by status, and a sense of shame. In contrast, societies located at the short-term pole prefer virtues related to the past and present, in particular respecting for tradition, protecting one’s “face,” and fulfilling social obligations. East Asian countries mostly have a long-term orientation, while Australia, United States, some Latin American, African, and Muslim countries can be identified as short-term orientated societies (Hofstede, 2001).

Indulgence versus restraint was originally extracted from the World Values Survey and adopted by Hofstede in his model (Hofstede et al., 2010). IVR reflects the extent to which a society responds to human basic needs. A high IVR score stands for a relatively weaker constrain of feelings and primitive human needs regarding the enjoyment of life and having fun (recreation, money spending, consumption, and sex), while a low IVR score stands for a relative stronger constrain of these needs through strict social norms (Hofstede et al., 2010). IVR is a big cultural dimension that covers various aspects of cultural phenomena and shows cross-temporal stability (Beugelsdijk et al., 2015). Maleki and de Jong (2014) conducted factor analysis on 20 cultural dimension scores with Varimax Rotation and found that IVR loaded on a factor on which other cultural dimensions did not load. American and Western European countries mostly have an indulgence orientation, while Eastern European and Asian countries tend to have a restraint orientation.

### Cultural Values and Prosocial Behavior

Culture values are important factors in determining an individual’s social behavior (Bond and Hofstede, 1989; Hofstede, 2001). Country-level differences in prosocial behavior and its influential factors have been investigated by recent studies, such as economic factors (Luria et al., 2015), religiosity (Guo et al., 2018), and perceived corruption and interpersonal trust (Smith, 2015). Apart from these findings, a handful of studies showed that national culture values can robustly account for country-level variance in prosocial behavior above economy and human development factors. Using country-level data from the WGI provided by the Charities Aid Foundation, and data for Hofstede’s national cultural values (Hofstede, 1980, 2001), Luria et al. (2015) found that at national level IND was positively correlated with prosociality, while power distance, uncertainty avoidance, and LTO were negatively correlated with prosociality. Luria et al. (2015) further found that the association of IND and prosociality was moderated by power distance and uncertainty avoidance. Specifically, in countries with low power distance and uncertainty avoidance, IND is more strongly associated with prosocial behavior. Winterich and Zhang (2014) also found a negative association between power distance and country-level prosocial behavior, when the effects of other four Hofstede’s cultural dimensions were controlled (Study 1). Then they conducted several follow up studies at individual level to investigate related mechanisms. Their individual level analysis confirmed the relationship between power distance and prosocial behavior at national level (Study 2), and further showed that the negative effect of power distance belief on charitable behavior was mediated by perceived responsibility to help others (study 3). However, the negative effect of power distance belief on prosocial behavior could be attenuated if charitable behavior supports uncontrollable versus controllable needs (study 4), and if communal versus exchange relationship norms were elicited (study 5). Drawing on datasets from Schwartz Values Survey (Schwartz, 2004) and Hofstede’s (2001) cultural dimensions, Smith (2015) found that all three types of prosocial behavior were associated with low in-group favoritism (i.e., collectivism) and low uncertainty avoidance.

The reasons why national cultural values influence prosocial behavior have also been addressed by previous researchers. Winterich and Zhang (2014) suggested that high power distance in a society could reduce individuals’ perceived responsibilities to aid the needy. Luria et al. (2015) argued that in societies with high power distance, individuals tended to accept hierarchical work patterns. High-status people are less likely to engage in formal and informal helping because they are reluctant to interact with low social status people. Luria et al. (2015) argued that in high IND societies people felt more personal responsibilities to help the needy, while in collectivist societies people tended to expect the governments and other collective mechanisms to assume responsibility for reducing income inequality and helping the poor. Knafo et al. (2009) argued that individuals in societies that endorse embeddedness values (in-group favoritism or collectivism) tended to prioritize the welfare of family and in-groups over their own, and are less concerned with the welfare of the strangers or the out-groups. Based on this analysis, Smith (2015) suggested that in-group favoritism in collectivist societies can lead to lower levels of WGI because its three components were more often directed toward out-groups or strangers rather than the immediate in-groups. Additionally, the negative association between uncertainty avoidance and prosocial behavior can be interpreted as the results of negative emotionality (e.g., anxiety and neuroticism). Specifically, uncertainty avoidance could lead to higher levels of neuroticism in a society (Taras et al., 2010), which in turn undermines prosocial tendency of the individuals in that society (Handy and Cnaan, 2007). In Luria et al.’s (2015) study, LTO was negatively associated with donating money and helping a stranger. They argued that in societies with a long-term orientation individuals tended to engage in future-oriented behavior such as planning and investing for the future, and were less likely to engage in unplanned behaviors such as donating money or helping a stranger incidentally.

### The Present Study

Culture is a collective level phenomenon containing variable values, beliefs, and practices across societies (Kitayama and Uskul, 2011). Individuals’ cognitive, emotional, and motivational functioning as well as scripted behavioral patterns can be systemically influenced by culture. Recently there is a growing interest in how culture influences human psychology and behavior (e.g., Kitayama and Uskul, 2011; Henrich, 2015). For example, drawing on 2007 database of the Trends in International Mathematics and Science Study (TIMSS) including more than 400,000 fourth- and eighth-grade students from 62 countries, Bergmüller (2013) found that individualism was positively associated with principal-reported aggressive student behavior, even after controlling the characteristics of school and country. A meta-analysis revealed that Hofstede’s cultural values are just as robust as personality traits, cognitive abilities, and demographics in predicting individual level organizational outcomes such as commitment and citizenship behaviors (Taras et al., 2010). These findings seem plausible at national level. For example, individualism is positively associated with innovation and life satisfaction, uncertainty avoidance is positively associated with neuroticism and corruption (Taras et al., 2010). Recent literature also has confirmed the influences of several Hofstede’s cultural dimensions on prosocial behavior at national level (Winterich and Zhang, 2014; Luria et al., 2015; Smith, 2015).

However, two newly constructed cultural dimensions, IVR and LTO, have not received sufficient interest they deserve. First, the link between IVR and prosocial behavior has never been investigated by previous literature. Second, though Luria et al. (2015) used LTO as a predictor of prosocial behavior, their dataset only included 66 countries. Data for LTO of 93 countries was available in 2010 (Hofstede et al., 2010). In 2013, the number of countries increased to 96^1^. Furthermore, the cultural values of a country may change over time (Sortheix et al., 2017). Recent literature suggests that, on average, all societies have become more individualistic and indulgent, and are experiencing a decrease in power distance (Taras et al., 2012; Beugelsdijk et al., 2015). But the effect of LTO on prosocial behavior might have not been soundly addressed. So it is necessary to use renewed data of cultural values. Additionally, Luria et al.’s (2015) data for prosocial behavior was derived from a Gallup survey conducted more than 10 years ago (i.e., 2006). Drawing on newly released datasets, this study is aiming to examine whether IVR and LTO influence prosocial behavior at national level, and whether their influences are moderated by IND.

Indulgence versus restraint reflects the extent to which human needs and feelings were constrained. Freedom, emotional expression, and happiness are encouraged in indulgent societies. (Hofstede et al., 2010; Maleki and de Jong, 2014). In indulgent societies human basic needs are more likely to be gratified. People are encouraged to express their emotions and enjoy life (Hofstede et al., 2010; Maleki and de Jong, 2014). Minkov (2009) found that there were greater percentages of happy people in indulgent relative to restraint societies. The reason is that indulgent societies impose less restriction on the enjoyment of life. Previous literature has indicated the importance of positive emotions in promoting prosocial engagement (Lennon and Eisenberg, 1987; Wiepking and Breeze, 2012). This may be due to the fact that positive emotions are associated with sensitiveness to the needs of others (Lennon and Eisenberg, 1987; Aknin et al., 2012). Therefore, we hypothesized that IVR is positively related to prosocial behavior (Hypothesis 1a).

Luria et al. (2015) assumed that “in societies with a long term orientation, people expect to have more interaction with others in the future and are consequently more willing to help others.” (p. 7). But this assumption has not been supported. In fact, people in high LTO societies prefer long-term plans, but prosocial behavior like helping strangers and donating money were usually spontaneous. Indeed WGI mostly concerns prosocial behavior toward strangers which was usually out of a long-term plan. Thus we hypothesized that LTO is negatively related to prosocial behavior, especially helping a stranger (Hypothesis 1b).

In cross-cultural psychology, IND is “a reliable and valid dimension of cultural differences” (Schimmack et al., 2005, p. 30). IND may be the most important cultural dimension that has received much more research interest than other cultural dimensions (Oyserman et al., 2002; Schimmack et al., 2005). Country level analysis has revealed systematic differences in emotional, cognitive, and social functioning in participants from individualist societies than those from collectivist societies (Oyserman et al., 2002; Kitayama and Uskul, 2011). For example, in individualist societies, individuals will experience intrinsic motivation if they have chance to make personal choices, while in collectivist societies individuals may also experience an internalized motivation if the choices are made by in-group others (Hagger et al., 2014). IND may be the most useful and most soundly established culture dimension in interpreting East-West differences (Oyserman et al., 2002; Triandis and Suh, 2002; Kitayama and Uskul, 2011; Bergmüller, 2013). Luria et al. (2015) proposed that culture values “are interdependent, and can be expected to interact in prediction of prosocial behaviors” (p. 8). And in their study, it was found that both power distance and uncertainty avoidance could moderate the relationship between IND and prosocial behavior. Hofstede (1985) (p. 352), also suggested that “different combinations of power distance and uncertainty avoidance lead to different implicit models in people’s minds.” Therefore, it seems feasible to explore the moderation effects of culture dimensions. In this study we are intended to explore whether (and how) the associations between IVR as well as LTO and prosocial behavior were moderated by IND, although there is insufficient literature to support our proposition.

To fully understand how Hofstede’s cultural values interact in influencing country-level prosociality, we also further explored whether (and how) the relationship between LTO and prosocial behavior was moderated by IVR.

## Materials and Methods

### Dependent and Independent Variables

We used the 2016 WGI as a general indicator of country-level prosocial behavior. WGI was published by the Charities Aid Foundation^2^ using data gathered by Gallup. Three components of WGI (i.e., donating, volunteering time, and helping a stranger) were measured each by an item asking the participants if they have given money to charity/volunteered for an organization/helped a stranger in the month previous to the survey. For each of these three questions, a percentage of participants who said yes were calculated. Then these three percentages were averaged within a country to form an aggregate score representing prosocial behavior at national level. If a country had missing data in the 2016 WGI, the missing value was replaced by the average score of the years available during 2010–2015. Previous studies showed that WGI can be used as a reliable and valid measure of prosocial behavior (e.g., Smith, 2015; Guo et al., 2018).

Scores for three cultural dimensions at country level were mainly drawn from Hofstede et al. (2010) with an update in 2013 on Hofstede’s official website^3^. In this study this dataset was further complemented by the scores calculated by Beugelsdijk et al.’s (2015). To investigate the stability of Hofstede’s culture values over time, Beugelsdijk et al. (2015) estimated the scores of Hofstede’s dimensions for two age groups using items drawn from World Values Survey (WVS)^4^. The estimated scores of LTO, IVR, and IND (Beugelsdijk et al., 2015) correlated with the original scores (Hofstede et al., 2010) at 0.94, 0.92, and 0.77, respectively. We averaged scores of the two age groups. Then we used these average scores as predictors and Hofstede’s original scores as dependent variables. The missing values in Hofstede’s original dataset were replaced by the predicted values in the regression equations. Finally data for IVR, LTO, and IND was available for 90, 89, and 94 countries, respectively. Finally there were 95 countries left in our dataset with scores for prosocial behavior and at least one cultural dimension.

### Control Variables

Based on previous findings that religion and economic factors (Luria et al., 2015; Guo et al., 2018) also influence country-level prosocial behavior (HDI; a composite statistic of life expectancy, education, and per capita income indicators), and religion (national religiosity) were used as control variables. National religiosity was taken from Guo et al. (2018). Based on WVS, they measured the religiosity of 96 countries across the world in terms of church attendance, prayer, deity importance, religion importance, and the proportion of religious people. Data for 2016 HDI was released by the United Nation^5^. Missing data of a country was replaced by the average score of other countries that are in the same continent (e.g., Europe, America, Asia, or Africa) as that country.

## Results

### Relationships Between Cultural Dimensions and Prosocial Behavior

Pearson correlations between IVR, IND, LTO, and WGI (including all three components) were shown in **Table 1**. Power distance, uncertainty avoidance, and masculinity versus femininity were also included in the correlation analysis in order to make a comparison with the results of previous studies. As **Table 2** illustrated, IVR was positively correlated with three indicators of prosocial behavior (for donating, *r* = 0.40; for volunteering, *r* = 0.52; for helping, *r* = 0.43; for WGI, *r* = 0.54*; p*s < 0.001), while LTO was only significantly negatively correlated with helping a stranger (*r* = -0.57*, p* < 0.001). And IND was significantly positively correlated with donating (*r* = 0.45*, p* < 0.001) and volunteering (*r* = 0.29*; p* < 0.01). This indicated that IVR may be the most important predictor of prosocial behavior among the three cultural dimensions. Among the three cultural dimensions, the correlation between IVR and LTO was negative (*r* = -0.45, *p* < 0.001), and the correlation of LTO with IND was positive (*r* = 0.28, *p* < 0.001), while the relationship between IVR and IND was insignificant. These results suggested that these three cultural dimensions are essentially different constructs that may each contribute uniquely to prosocial behavior.

**Table 1:** Correlations among research variables.

	*M*	*SD*	PD	UA	MF	IVR	LTO	IND	Donating	Volunteering	Helping	WGI	Religion	HDI
PD	60.36	19.23	1											
UA	67.13	21.93	0.20	1										
MF	49.56	20.18	0.14	–0.03	1									
IVR	45.92	22.60	–0.29	–0.04	0.04	1								
LTO	44.28	23.64	–0.04	–0.09	0.07	–0.45***	1							
IND	39.97	22.40	–0.68**	–0.22	0.05	0.14	0.28***	1						
Donating	33.18	19.25	–0.50**	–0.44**	–0.17	0.40***	0.04	0.45***	1					
Volunteering	21.01	10.39	–0.28**	–0.25**	–0.01	0.52***	–0.20	0.29**	0.64***	1				
Helping	50.53	12.05	–0.21**	–0.23**	–0.15	0.43***	–0.57***	–0.02	0.32**	0.48***	1			
WGI	34.98	11.29	–0.44**	–0.40**	–0.14	0.54***	–0.23*	0.34**	0.88***	0.84***	0.68***	1		
Religion	–0.44	4.64	0.44**	0.13	–0.06	0.06	–0.62***	–0.55***	–0.35*	–0.00	0.34**	–0.08	1	
HDI	0.77	0.13	–0.52**	0.10	0.21*	0.41***	0.60**	0.53***	0.20		–0.16	0.31**	–0.73***	1


*Note: PD, power distance; UA, uncertainty avoidance; MF, masculinity versus femininity; ^∗^*p* < 0.05. ^∗∗^*p* < 0.01. ^∗∗∗^*p* < 0.001.*

**Table 2:** Predictive values of cultural dimensions (*N* = 61).

	WGI		Donating		Volunteering		Helping	
	*β*	*t*	*β*	*t*	*β*	*t*	*β*	*t*
Religion	0.25	1.87	0.15	1.14	0.26	1.71	0.19	1.24
HDI	0.41**	3.04	0.52***	3.86	0.28	1.77	0.16	1.06
UA	–0.43***	–4.56	–0.45***	–4.79	–0.29**	–2.74	–0.26*	–2.58
IVR	0.37**	3.60	0.21*	2.02	0.39**	3.28		
LTO							–0.51***	–4.10
PD							–0.27*	–2.14
*R^2^*	0.51^∗∗∗^		0.52^∗^		0.35^∗∗^		0.46^∗^	


*Note: The regression coefficients and t-values for the excluded variables were presented in parentheses; PD, power distance; UA, uncertainty avoidance; MF, masculinity versus femininity; ^∗^p < 0.05. ^∗∗^p < 0.01. ^∗∗∗^p < 0.001.*

Consistent with Luria et al. (2015), power distance and uncertainty avoidance were significantly and negatively correlated with prosocial behavior (power distance: for donating, *r* = -0.50; for volunteering, *r* = -0.28; for helping, *r* = -0.21; for WGI, *r* = -0.44*, p*s < 0.01; uncertainty avoidance: for donating, *r* = -0.44; for volunteering, *r* = -0.25; for helping, *r* = -0.23; for WGI, *r* = 0.40*; p*s < 0.01), while masculinity versus femininity was not associated with prosocial behavior.

Hierarchical multiple regression was used to analyze the unique contribution of each cultural dimension. Religion and HDI as the controls were entered in Step 1, and six cultural dimensions were added in the regression model using a stepwise method in Step 2 (**Table 2**). This method enables us to find the variables whose predictive values cannot be substituted by other variables. **Table 2** showed that (1) when WGI, donating, and volunteering were outcomes, the effects of uncertainty avoidance and IVR were significant (for uncertainty avoidance, *b* = -0.43, -0.45, -0.29, *t* = -4.56, -4.79, -2.74, respectively, *p*s < 0.01; for IVR, *b* = 0.37, 0.21, 0.39, *t* = 3.60, 2.02, 3.28, respectively, *p*s < 0.05), with other four cultural dimensions providing no more predictive power; (2) when helping a stranger was the outcome, the effects of LTO, uncertain avoidance, and power distance were significant (*b* = -0.51, *t* = -4.10, *p* < 0.001; *b* = -0.26, *t* = -2.58, *p* < 0.05; *b* = -0.27, *t* = -2.14, *p* < 0.05, respectively). These findings suggested that the two newly developed cultural dimensions (especially IVR) in Hofstede’s model are useful in predicting country-level prosociality. Uncertainty avoidance was the only cultural dimension that was predictive of WGI and its three components above other cultural dimensions, showing that it has particular importance in influencing prosocial behavior. Surprisingly, IND and religion did not exert significant influence on prosocial behavior. This is inconsistent with Luria et al. (2015) and Smith (2015). Including IVR in our regression models may have made the differences.

### Moderation Analyses

Then we examined whether these three cultural dimensions interact with each other in predicting prosocial behavior. The model 1 of PROCESS (a SPSS macro, Hayes, 2013) was employed to estimate the moderation models using 5000 bootstrap samples. Results were shown in **Tables 3–5**.

**Table 3:** IVR Interacted with IND in predicting prosocial behavior (*N* = 88).

	WGI			Donating			Volunteering			Helping		
	*β*	*SE*	*t*	*β*	*SE*	*t*	*β*	*SE*	*t*	*β*	*SE*	*t*
IVR	0.55	0.08	6.66***	0.38	0.08	4.52***	0.51	0.10	5.27***	0.50	0.09	5.64***
IND	0.10	0.10	0.97	0.04	0.10	0.35	0.21	0.12	1.77	0.04	0.11	0.35
IVR × IND	0.43	0.08	5.35***	0.42	0.08	5.18***	0.25	0.09	2.71**	0.32	0.09	5.64***
Religion	0.18	0.12	1.56	0.03	0.12	0.82	0.17	0.14	1.24	0.33	0.13	2.65**
HDI	0.21	0.12	1.66	0.37	0.13	2.92**	0.05	0.15	0.36	–0.06	0.13	–0.42
*R^2^*	0.55^∗∗∗^			0.53^∗∗∗^			0.39^∗∗∗^			0.43^∗∗∗^		


*Note: ^∗∗^p < 0.01. ^∗∗∗^*p* < 0.001.*

**FIGURE 1:**
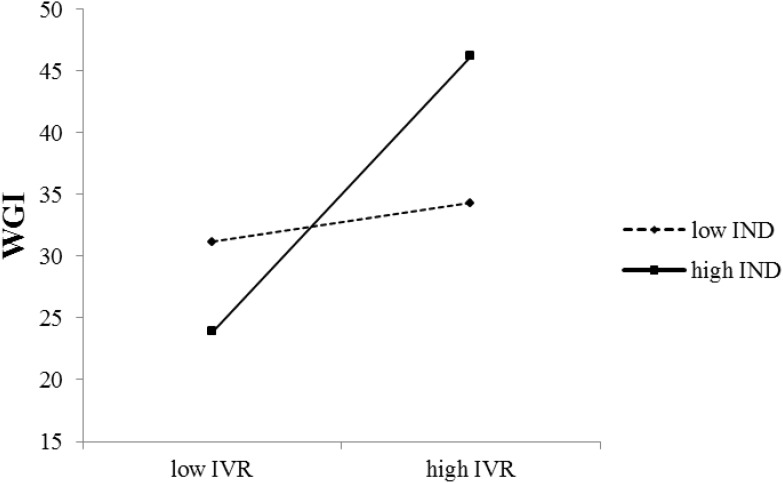
The Effect of IVR on WGI, Moderated by IND.

As shown in **Table 3**, the interaction term of IVR and IND could significantly predict WGI and its three components, suggesting that the relationship between IVR and prosocial behavior was moderated by IND. Simple slope analysis showed that in low IND societies (low = 1 SD below the centered mean; high = 1 SD above the centered mean) IVR did not influence WGI [*β* = 0.14, *t* = 1.35, *p* = 0.18, 95% confidence interval, CI = (-0.06, 0.35)], whereas in high IND societies the influence of IVR on WGI was significant [*β* = 0.99, *t* = 7.91, *p* < 0.001, 95% CI = (0.74, 1.24)], see **Figure 1**. Simple slope analysis revealed similar findings when the outcomes were donating [Low IND: *β* = -0.02, *t* = -0.22, *p* = 0.82, 95% CI = (-0.23,0.18); High IND: *β* = 0.81, *t* = 6.40, *p* < 0.001, 95% CI = (0.56, 1.06)], and helping a stranger [Low IND: *β* = 0.19, *t* = 1.78, *p* = 0.08, 95% CI = (-0.02, 0.42); High IND: *β* = 0.83, *t* = 6.13, *p* < 0.001, 95% CI = (0.56, 1.10)]. Furthermore, the influence of IND on the relationship of volunteering with IVR is stronger in high IND countries [*β* = 0.77, *t* = 5.26, *p* < 0.001, 95% CI = (0.48, 1.07)] than in Low IND countries [*β* = 0.27, *t* = 2.20, *p* = 0.03, 95% CI = (0.03, 0.51)]. These results suggested that indulgence in a society generally has a positive effect on prosocial behavior, but this effect is much stronger in individualist societies than in collectivist societies.

**FIGURE 2:**
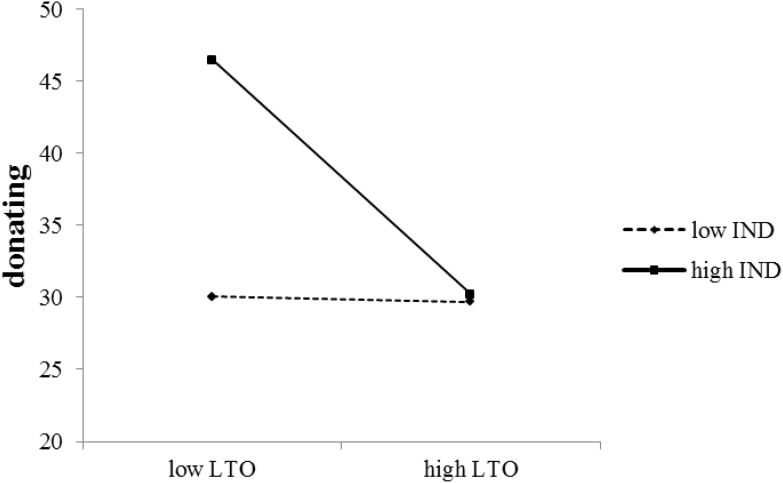
The Effect of LTO on Donating, Moderated by IND.

In **Table 4**, the significant interaction between LTO and IND showed that the relationship between LTO and donating can be influenced by IND. Simple slope analysis showed that in low IND societies LTO did not influence donating [*β* = -0.01, *t* = -0.06, *p* = 0.95, 95% CI = (-0.31, 0.29)], while in high IND societies the influence of LTO on donating was significantly negative (*β* = -0.42, *t* = -0.16, *p* = 0.01, 95% CI = (-0.73, -0.10)], see **Figure 2**.

**Table 4:** LTO Interacted with IND in predicting prosocial behavior (*N* = 87).

	WGI			Donating			Volunteering			Helping		
	*β*	*SE*	*t*	*β*	*SE*	*t*	*β*	*SE*	*t*	*β*	*SE*	*t*
LTO	–0.40**	0.12	–3.35	–0.20	0.12	–1.77	–0.29*	0.13	–2.25	–0.55***	0.11	–4.92
IND	0.31*	.12	2.23	0.22	0.12	1.91	0.35**	0.13	2.70	0.20	0.11	1.77
LTO × IND	–0.20	0.10	–1.93	–0.21*	0.10	–2.05	–0.15	0.11	–1.35	–0.12	0.10	–1.27
Religion	0.11	0.16	0.66	0.02	0.15	0.12	0.18	0.17	1.01	0.13	0.15	0.87
HDI	0.34*	0.15	2.26	0.46**	0.15	3.19	0.21	0.16	1.27	0.03	0.14	0.21
*R^2^*	0.32^∗∗∗^			0.36^∗∗∗^			0.22^∗∗∗^			0.36^∗∗∗^		


*Note: ^∗^p < 0.05. ^∗∗^p < 0.01. ^∗∗∗^p < 0.001.*

**FIGURE 3:**
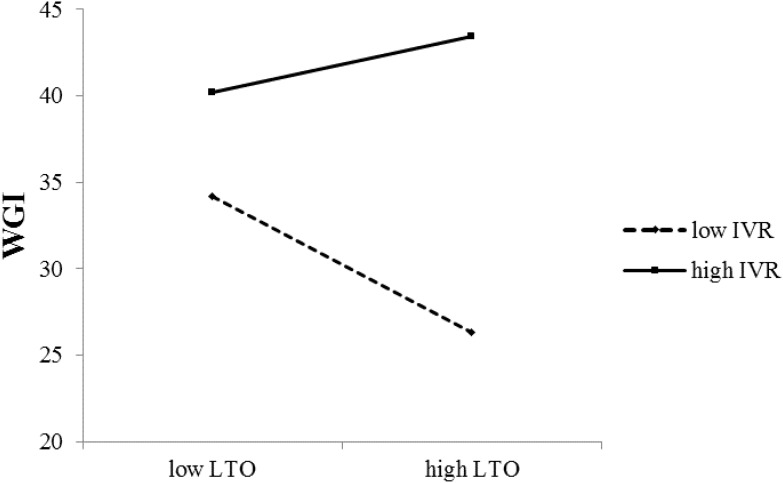
The Effect of LTO on WGI, Moderated by IVR.

**Table 5** showed that the interaction effect of LTO and IVR in predicting prosocial behavior was also significant, showing that the relationship between LTO and prosocial behavior (excepting volunteering) was moderated by IVR. Simple slope analysis showed that LTO had a negative influence on WGI in low IVR societies [*β* = -0.35, *t* = -2.32, *p* = 0.02, 95% CI = (-0.65, -0.05)], while the influence of LTO on WGI is insignificant in high IVR societies [*β* = 0.14, *t* = 0.80, *p* = 0.43, 95% CI = (-0.21, 0.50)], see **Figure 3**. Similar results were found when the outcome was helping a stranger [Low IVR: *β* = -0.56, *t* = -3.77, *p* < 0.001, 95% CI = (-0.86, -0.26); High IVR: *β* = -0.17, *t* = -0.98, *p* = 0.33, 95% CI = (-0.52, 0.18)]. But both in low and high IVR societies, influences of LTO on donating money were not significant [Low IVR: *β* = -0.22, *t* = -1.50, *p* = 0.14, 95% CI = (-0.51, 0.07); High IVR, *β* = 0.27, *t* = 1.53, *p* = 0.13, 95% CI = (-0.08, 0.61)].

**Table 5:** LTO Interacted IVR with in predicting prosocial behavior (*N* = 88).

	WGI			Donating			Volunteering			Helping		
	*β*	*SE*	*t*	*β*	*SE*	*t*	*β*	*SE*	*t*	*β*	*SE*	*t*
LTO	–0.10	0.14	–0.73	0.03	0.13	0.19	0.05	0.15	0.31	–0.36**	0.14	–2.68
IVR	0.51***	0.12	4.29	0.40**	0.12	3.42	0.55***	0.13	4.25	0.32**	0.12	2.76
IVR × LTO	0.24**	0.09	0.27	0.24**	0.09	2.71	0.12	0.10	1.21	0.19*	0.09	2.13
Religion	–0.01	0.15	–0.06	–0.08	0.15	–0.58	0.08	0.16	0.47	0.05	0.15	0.31
HDI	0.24	0.14	1.7	0.39**	0.14	2.76	0.13	0.16	0.81	–0.06	0.14	–0.42
*R^2^*	0.39^∗∗∗^			0.42^∗∗∗^			0.29^∗∗∗^			0.39^∗∗∗^		


*Note: ^∗^p < 0.05. ^∗∗^p < 0.01. ^∗∗∗^p < 0.001.*

## Discussion

It has been suggested that the fundamental psychological processes of an individual, such as cognition, judgment, evaluation, emotion, etc, can be systematically influenced by the culture values of a society (Kitayama and Uskul, 2011). Consistent with Hypothesis 1a, this study revealed a positive association between IVR and country-level prosociality. IVR may be the most important cultural dimension accounting for variance in prosocial behavior across nations. IVR indicates the extent to which gratification of human basic needs and feelings is free or restrained. Indulgent societies encourage people to express their emotions and enjoy life (Hofstede et al., 2010; Maleki and de Jong, 2014). Thus people have a lot of freedom to make personal choice and control over their life, and tend to experience greater happiness (Hofstede et al., 2010; Beugelsdijk et al., 2015). Higher levels of prosocial behavior in indulgent societies may be partly accounted for by higher levels of positive emotions of residents in these societies. Emotions play an important role in charitable giving (Aknin et al., 2012; Wiepking and Breeze, 2012). People with positive emotions respond better to the needs of others and tend to take helpful actions. For example, one study in children found that emotional expressiveness was predictive of children’s empathy, which in turn promoted children’s prosocial behavior (Roberts and Strayer, 1996). Another mechanism that indulgence promotes prosocial behavior may be the freedom in making personal choice and control. Previous literature suggested that self-determination is conductive to intrinsically motivated prosocial behavior. For example, Gagné (2003) found that autonomy orientation and autonomy support from significant others could lead to the satisfactions of three basic psychological needs (i.e., autonomy, competence, and relatedness), which were conductive to engagement in prosocial behavior. More importantly, autonomous motivation can increase the emotional benefits derived from prosocial behavior (Weinstein and Ryan, 2010). We infer that in indulgent societies, there may be a positive feedback loop between prosocial behavior and happiness. In other words, positive emotions lead to more intrinsically motivated prosocial behavior, which further enhances the happiness of those who performed it (Aknin et al., 2012). However, in countries of low IVR, people’s basic desires and emotions are constrained by social norms, resulting in relatively stronger negative emotions and consequently less prosocial behavior.

Long-term versus short-term orientation is also an important cultural dimension in influencing prosocial behavior. In this study, the negative association between LTO and helping a stranger was rather strong, which is partly consistent with Hypothesis 1b. A possible explanation is that, in short-term orientated societies service to others is considered as an important goal, while in long-term orientated societies thrift and perseverance are considered as important goals (Hofstede et al., 2010). Luria et al. (2015) suggested that individuals in long-term orientated societies may be more likely to engage in planned behaviors, and are less likely to help others by accident. Another explanation may be that, in Long-term orientated societies people are more likely to invest in long-term social networks or interpersonal relations with acquaintances (Hofstede and Minkov, 2010), and are less likely to interact with strangers.

In this study the effect of IND on prosocial behavior was partialed out by IVR, LTO, religion, and HDI, though it has been revealed by previous studies (Luria et al., 2015; Smith, 2015). The diminished effect may be attributed to the fact that HDI was used as a control. IND and economic indicators were highly correlated at the national level (Stojcic et al., 2016), and the calculation of HDI involves economic factor. In addition, we also found a strong correlation between IND and HDI at national level, which was consistent with Schimmack et al. (2005). Interestingly and surprisingly, the moderation effect of IND was found in the relationships of prosocial behavior with IVR and LTO.

The results showed that IND played a moderating role in the relationship between IVR and prosocial behavior. High IND in a country could strengthen the relationship of IVR with WGI and its components. That is, the IVR-prosociality association was stronger in high IND countries than in low IND countries. This may be explained by the fact that components of WGI are prosocial behavior primarily directed toward out-groups/strangers. In collectivist societies, IVR may promote prosocial behavior toward in-groups, while in individualist societies IVR may promote prosocial behavior toward out-groups or strangers (Smith, 2015).

Individualism versus collectivism also played a moderating role in the relationship between LTO and prosocial behaviors. A significant interaction between LTO and IND in predicting money donation indicated that the negative LTO-donation association tended to be stronger in individualist societies than in collectivist societies. Collectivism emphasizes interpersonal relatedness and commitment to groups or the society (Luria et al., 2015). The negative effect of LTO on prosocial behavior may somewhat be weakened by collectivism because social ties are strengthened in collectivist (low IND) societies (Finkelstein, 2010). Therefore, in societies not emphasizing the importance of service to others (high LTO) and interpersonal relatedness (high IND), less prosocial behavior is expectable.

Furthermore, a moderating effect of IVR in the LTO-prosociality relationship also was found. The effect of LTO on prosocial behavior was significant in low IVR societies, but insignificant in high IVR societies. Long-term orientated societies encourage thrift and perseverance and discourage service to others. One manifestation of LTO is a conservative attitude toward money that may deter prosocial behavior (Wiepking and Breeze, 2012). These negative effects on prosocial behavior could be attenuated by indulgent values in a society where the gratification of human basic needs and feelings are encouraged (Aknin et al., 2012; Geenen et al., 2014).

### Strengths and Limitations

Drawing on the newly published datasets and a relatively larger sample size, this study explored the effects of two newly established cultural dimensions in Hofestede’s model (IVR and LTO) on prosocial behavior that have received little interest in previous studies. We found that the effects of LTO and IVR on prosocial behavior were moderated by IND. These findings have made a significant progress in explaining why prosocial behavior varies across cultures. However, this study also has limitations.

Previous literature suggests that WGI is a reliable measure of prosocial behavior (Daniel et al., 2015; Smith, 2015), but what it measures is mainly behavior toward out-groups or strangers. Zwick and Fletcher (2014) proposed eight levels of altruism in a hierarchical order that progressively involve an expanding sense of the self. Self-interest (level 1) is the basis of this hierarchy, which can be progressively extended to kin altruism (level 2), interaction-based altruism (level 3), group altruism (level 4), species altruism (level 5), sentience altruism (level 6), life altruism (level 7), and being altruism (level 8). Each type of altruism is important for human society. Kin altruism and interaction-based altruism are the keys to cooperative relations in kin and other small groups. Group altruism, or group solidarity, is also important in maintaining harmonious relations in organizations, communities, ethnic groups, and even nations. What WGI stressed is perhaps primarily the altruism of human species that encourages people to treat out-groups or strangers as their in-groups (Smith, 2015). So whether Hofstede’s cultural model can predict other types of prosocial behavior is an interesting question merits deeper investigation. Furthermore, WGI provided by Gallup only concerns the frequencies of donating, volunteering, and helping. It is obvious that the frequency of donation is quite different from the amount of money donated (Petrovski, 2017). For example, Apinunmahakul (2014) reported that in 2011 the Thailand participants averagely gave less money than the participants in United States, but in Gallup’s WGI report the donation percentage score of Thailand (85%) was higher than that of the United States (65%).

### Applications and Future Directions

Increasing prosocial behavior in a society is beneficial not only for social harmony and solidarity (Zwick and Fletcher, 2014), but also for the psychological well-being of the actors (Weinstein and Ryan, 2010; Aknin et al., 2013; Geenen et al., 2014). Consistent with previous studies (e.g., Winterich and Zhang, 2014; Luria et al., 2015; Smith, 2015), our results confirmed the impact of cultural dimensions on prosocial behavior. This may be particularly valuable as many societies are experiencing change in values during the processes of modernization and globalization in recent years (Beugelsdijk et al., 2015). Thus, our findings are useful for policy makers to take appropriate actions to increase prosocial behavior in a society. First, we propose that emotional expressiveness, enjoying life, personal choice and control should be more strongly encouraged in social settings (e.g., organizational and educational). In these settings, values such as service to others, interpersonal relatedness, social spending and consumption should also be encouraged. Additionally, under the influence of globalization, interactions among ethnic groups and societies are becoming more and more frequently. People are encouraged to treat all humans as their “in-groups.”

This study showed that the interactions among IVR, LTO, and IND also have significant effects on prosocial behavior. Nevertheless, we have not provided sound explanations for these interactions. For example, why the relationship between IVR and prosocial behavior is stronger in high versus low IND countries? These questions are expected to be addressed elaborately by future research.

## Conclusion

In this study IVR was the only cultural dimension contributing to all three types of prosocial behavior. Its influence remained robust after the effects of other cultural dimensions were controlled. This is consistent with previous findings that prosocial behavior is mainly motivated by emotion (Aknin et al., 2012; Wiepking and Breeze, 2012). Surprisingly, the effect of IND on prosocial behavior diminished when other variables were introduced into the regression equations. This is consistent with previous propositions that IND is not always the most important cultural dimension (Taras et al., 2010).

Long-term versus short-term orientation has exerted a strong negative influence on helping a stranger, suggesting that devaluing the importance of serving others and endorsement of thrift and perseverance in a society can have a negative impact on building prosocial ethos. Moderation analyses have also revealed several valuable findings: (1) IVR is more strongly conductive to prosocial behavior in individualist societies than in collectivist societies; (2) low IND in a society (e.g., emphasizing interpersonal relatedness and commitment to groups or a society) could weaken the negative effect of LTO on donating; (3) high IVR in a society (e.g., encouraging the gratification of human basic needs and feelings) could attenuate the negative effect of LTO on prosocial behavior.

## Author Contributions

QG designed the study and wrote the manuscript. ZL and XL wrote the manuscript and collected the data. ZL and XQ collected and analyzed the data under the supervision of QG. QG, ZL, and XQ revised the manuscript, and replied to comments.

## Conflict of Interest Statement

The authors declare that the research was conducted in the absence of any commercial or financial relationships that could be construed as a potential conflict of interest.

## Footnotes

1 www.Geerthofstede.com

2 www.cafonline.org

3 www.Geerthofstede.com

4 http://www.worldvaluessurvey.org

5 http://report.hdr.undp.org

## Abbreviations

HDI: human development index
IND: individualism versus collectivism
IVR: indulgence versus restraint
LTO: long-term versus short-term orientation
WGI: world giving index
